# New Strategies to Direct Therapeutic Targeting of PML to Treat Cancers

**DOI:** 10.3389/fonc.2013.00124

**Published:** 2013-05-17

**Authors:** Kamil Wolyniec, Dennis A. Carney, Sue Haupt, Ygal Haupt

**Affiliations:** ^1^Tumour Suppression Laboratory, Peter MacCallum Cancer CentreEast Melbourne, VIC, Australia; ^2^Sir Peter MacCallum Department of Oncology, The University of MelbourneParkville, VIC, Australia; ^3^Department of Haematology, Peter MacCallum Cancer CentreEast Melbourne, VIC, Australia; ^4^Department of Pathology, The University of MelbourneParkville, VIC, Australia; ^5^Department of Biochemistry and Molecular Biology, Monash UniversityClayton, VIC, Australia

**Keywords:** PML, E6AP, BMK1, CK2, KLHL20, tumour suppression, targeted anti-cancer therapy, small molecule inhibitors

## Abstract

The tumor suppressor function of the promyelocytic leukemia (PML) protein was first identified as a result of its dysregulation in acute promyelocytic leukemia, however, its importance is now emerging far beyond hematological neoplasms, to an extensive range of malignancies, including solid tumors. In response to stress signals, PML coordinates the regulation of numerous proteins, which activate fundamental cellular processes that suppress tumorigenesis. Importantly, PML itself is the subject of specific post-translational modifications, including ubiquitination, phosphorylation, acetylation, and SUMOylation, which in turn control PML activity and stability and ultimately dictate cellular fate. Improved understanding of the regulation of this key tumor suppressor is uncovering potential opportunities for therapeutic intervention. Targeting the key negative regulators of PML in cancer cells such as casein kinase 2, big MAP kinase 1, and E6-associated protein, with specific inhibitors that are becoming available, provides unique and exciting avenues for restoring tumor suppression through the induction of apoptosis and senescence. These approaches could be combined with DNA damaging drugs and cytokines that are known to activate PML. Depending on the cellular context, reactivation or enhancement of tumor suppressive PML functions, or targeted elimination of aberrantly functioning PML, may provide clinical benefit.

## Introduction

The promyelocytic leukemia (PML) gene was initially described in the pathogenesis of Acute Promyelocytic Leukemia (APL), where it fuses with the Retinoic Acid Receptor α (RARα) gene as a consequence of the chromosomal translocation *t*(15;17). The resultant PML-RARα fusion oncoprotein acts in a dominant negative fashion over wild type (wt) PML, as reiterated in a mouse model (Rego and Pandolfi, 2001); where malignancy manifests from a differentiation blockage of granulocyte precursors which is compounded by an enhanced self-renewal (de Thé and Chen, 2010). All-trans retinoic acid (ATRA) and arsenic trioxide (As_2_O_3_) were empirically identified to provoke a profound therapeutic response against APL, before the molecular pathogenesis of the disease was established (Huang et al., 1988; Borrow et al., 1990; de Thé et al., 1990; Sun et al., 1992) and unravelling intricacies of their mode of action toward PML-RARα is ongoing. The intense research that followed the landmark discovery linking PML to APL pathogenesis, revealed that PML acts as a tumor suppressor in many other cancer types and is a master regulator of major cellular processes.

Promyelocytic leukemia comprises multiple isoforms, which predominantly localize to the nucleus (Bernardi and Pandolfi, 2007; Carracedo et al., 2011). In response to various stress signals, PML forms distinct matrix-associated structures in the nucleoplasm, called PML nuclear bodies (NBs) (otherwise known as PML-NBs, POD, ND10) (Bernardi and Pandolfi, 2007; Carracedo et al., 2011). Over 70 proteins are known to co-localize with PML in the NBs, mainly in a transient manner (Bernardi and Pandolfi, 2007; Carracedo et al., 2011). PML-NBs mediate the post-translational modification of target proteins and in a spatio-temporal manner coordinate a diverse range of specific cellular functions, including gene transcription, DNA repair, apoptosis, senescence, and anti-viral response (Dellaire and Bazett-Jones, 2004; Geoffroy and Chelbi-Alix, 2011). In APL, the expression of PML-RARα is associated with numerous, disorganized nuclear microstructures instead of the normal PML-NBs (Koken et al., 1994; Weis et al., 1994).

A well-defined downstream effector pathway of PML (Salomoni et al., 2008, 2012) involves the key tumor suppressor p53. In response to stress, PML promotes the activation and stabilization of p53 by protecting it from its major inhibitor Mdm2, and facilitating key post-translational modifications (Louria-Hayon et al., 2003; Bernardi et al., 2004; Alsheich-Bartok et al., 2008). Intriguingly, PML is also a transcriptional target of p53 (de Stanchina et al., 2004), implying that these two important tumor suppressors impact on each other through a positive regulatory loop. However, transcription is not the major dictator of altered PML levels (Gurrieri et al., 2004a) and in this review, we scrutinize our current knowledge regarding PML regulation. We suggest that the careful analysis of key upstream molecules that act upon PML in different cancer types will be a strategic approach toward rationally defining targets for the design of specific anti-cancer therapies with a capacity to restore functional PML.

## Loss of PML Function Promotes Tumorigenesis

The original studies by Koken et al. (1995) and Terris et al. (1995) revealed that PML expression is altered during the process of oncogenic transformation. In the subsequent studies PML protein levels were identified to be down-regulated (complete or partial loss) in a wide spectrum of human cancers, beyond APL, including additional hematological neoplasms: non-Hodgkin lymphomas (77%), carcinomas of the: prostate (92%), lung (58%), colon (47%); and breast (53%); tumors of the central nervous system (CNS; 73%) and germ cells (85%) (Gurrieri et al., 2004a); stomach (Lee et al., 2007), lung small cells (Zhang et al., 2000); and sarcomas of the soft tissues (Vincenzi et al., 2010). Pertinently, downregulation of PML is frequently associated with increased tumor grade and highly aggressive disease in some tumor types, e.g., prostate and breast adenocarcinomas (Rego and Pandolfi, 2001). PML tumor suppressive functions have been validated in a number of genetically engineered mouse cancer models. PML deficiency in the context of PTEN+/− mice, resulted in the invasive adenocarcinoma of the colon (Trotman et al., 2006). Dosage-dependent PML loss correlated with the number and size of colonic polyps. Further, the tumor burden and aggressiveness of KrasG12D-induced non-small cell lung cancer (NSCLC) was significantly increased in the absence of PML (Scaglioni et al., 2006). Most recently, PML tumor suppressive capacity was demonstrated in a mouse model of B-lymphoma driven by c-Myc (Wolyniec et al., 2012b) and in the context of mutant p53 (Haupt et al., 2013). These fundamental *in vivo* studies, together with detailed molecular analysis *in vitro*, have revealed major roles of PML in the induction of apoptosis and cellular senescence.

## Multiple Mechanisms of PML Regulation

**Figure 1:**
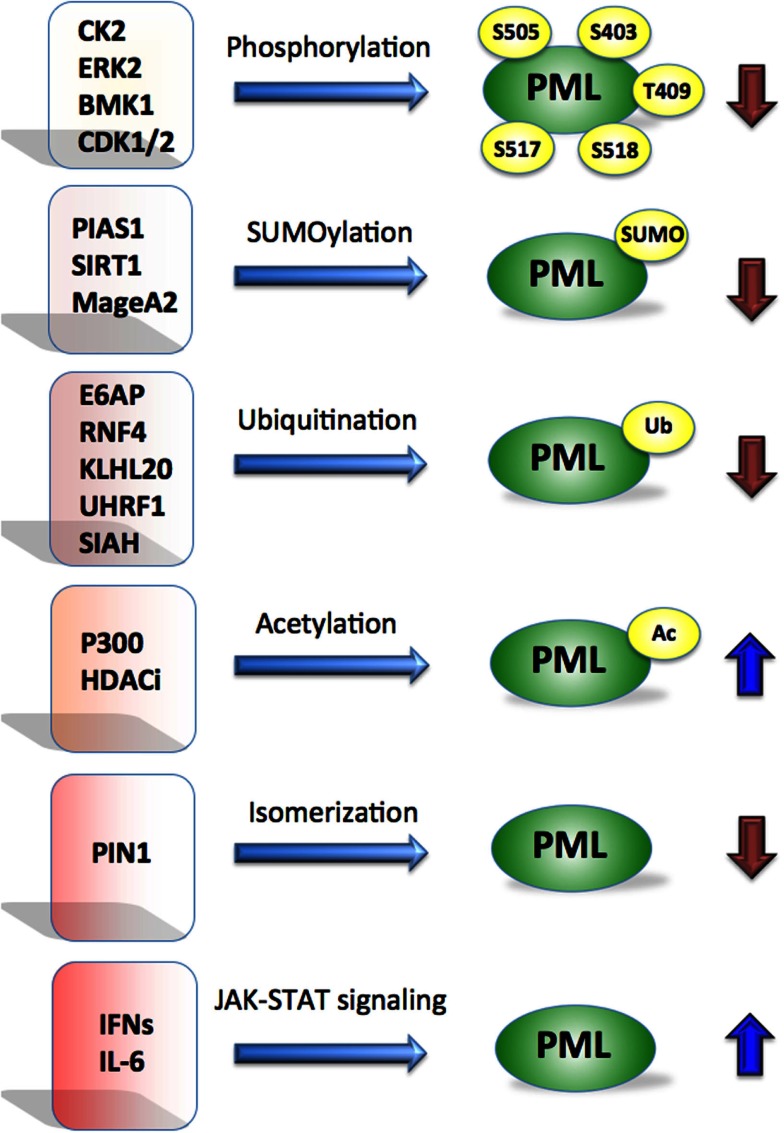
**Promyelocytic leukemia is regulated by multiple mechanisms**. Post-translational modifications such as phosphorylation, SUMOylation, ubiquitination, acetylation, isomerization by indicated proteins affect PML stability. Cytokines such as IFNs and IL-6 are known to activate PML at the transcriptional level via JAK-STAT signaling. Blue arrows indicate positive and brown negative effect on PML.

Loss of PML protein frequently occurs in cancers without correlation to PML mRNA levels, or gene mutation, but rather at a post-translational level (Gurrieri et al., 2004a,b). Importantly, proteasome inhibitor treatment of selected tumor cell lines lacking detectable PML levels (Gurrieri et al., 2004a), was able to restore PML expression. This provided a rationale for restoration of PML expression at the protein level. Molecular pathways that promote PML degradation are therefore potential targets for its restoration. Further, PML degradation is regulated by post-translational modifications including ubiquitination, phosphorylation, acetylation, SUMOylation, and isomerization (Figure 1); and each of these pathways has been implicated in various types of cancer (Table 1) and offers therapeutic potential.

**Table 1:** **Summary of PML modifications implicated in various human cancers**.

Type of PML modification	Cancer type	PML gain (+) or loss(−)	Reference	Section in the text
Ubiquitination by E6AP	B-cell lymphoma	−	Wolyniec et al. (2012b)	Section “Ubiquitination”
Phosphorylation by CK2	NSCL cancer	−	Scaglioni et al. (2006)	Section “Phosphorylation”
SUMOylation by PIAS1	NSCL cancer	−	Rabellino et al. (2012)	Section “SUMOylation”
Ubiquitination by KLHL20	Prostate cancer	−	Yuan et al. (2011)	Section “Ubiquitination”
Phosphorylation by ERK2/Pin1	Breast cancer	−	Lim et al. (2011)	Section “Phosphorylation”
Unknown mechanism	Breast cancer	+	Carracedo et al. (2012)	Section “Targeting GOF PML Activities in Non-Hematopoietic Malignancies”
Mutant p53 context	Colon cancer	+	Haupt et al. (2009)	Section “Targeting GOF PML Activities in Non-Hematopoietic malignancies”

*Reference to the section in the text and to the original publication is listed*.

### Ubiquitination

The finding that PML expression is largely regulated in cancer cells at the level of proteasomal degradation (Gurrieri et al., 2004a) triggered a search for the relevant E3 ligases that regulate the ubiquitination and subsequent degradation of PML in normal and cancer cells. In cancer cells the normal regulation of PML turnover is likely to become corrupted leading to destabilization of PML as a mechanism of evading tumor suppression (Chen et al., 2012b). E3 ligases able to promote the degradation of PML-RARα are also of obvious therapeutic value.

In a search for the key E3 ligase of PML we found that the E6-associated protein (E6AP) is a physiological E3 ubiquitin ligase of PML, and showed increased PML expression in multiple tissues of E6AP KO mice. One of the functional implications of this finding was that lymphoid cells derived from E6AP deficient mice were more susceptible to DNA-damage induced apoptosis, associated with enhanced accumulation of PML-NBs (Louria-Hayon et al., 2009). A more direct demonstration linking the E6AP-PML axis to cancer was evident in our recent study of Myc-driven B-cell lymphoma. We found that a loss of one E6AP allele was sufficient to attenuate B lymphomagenesis through the restoration of PML expression and induction of cellular senescence. Importantly, E6AP levels were elevated and associated with PML downregulation in more than half of the human B-cell lymphomas examined (Wolyniec et al., 2012b).

Another E3 ligase that is frequently elevated in various cancers is UHRF1 (ubiquitin-like with PHD and RING finger domains). UHRF1 was demonstrated to target PML for degradation by mediating its polyubiquitination in human umbilical vein endothelial cells (HUVEC) and cancer cell lines (Guan et al., 2012). The ubiquitin ligase ring finger protein 4 (RNF4) selectively targets poly-SUMOylated PML (see SUMOylation) (Lallemand-Breitenbach et al., 2008; Tatham et al., 2008). This process is a key mechanism of action of As_2_O_3_ resulting in the degradation of PML and PML-RARα in APL (see Targeting PML-RARα to Treat Acute Promyelocytic Leukemia). In addition to proteasomal degradation, autophagy also mediates the degradation of PML-RARα induced by ATRA and As_2_O_3_ (Boe and Simonsen, 2010). The ubiquitin-binding adaptor protein p62/SQSTM1 recognizes specific poly-ubiquitinated proteins including PML-RARα and directs them to autophagosomes for degradation (Pankiv et al., 2007; Wang et al., 2011).

*In vitro* studies have demonstrated that the RING finger E3 ligase SIAH-1/2 binds the coil–coil domain of PML, via its substrate-binding domain (SBD), and promotes the proteasomal degradation of PML and PML-RARα (Fanelli et al., 2004). An interesting study by Yuan et al., revealed a crucial role for the substrate adaptor protein KLHL20 of the Cullin 3-based ubiquitin ligase, in the regulation of PML in response to hypoxia, during tumor progression of prostate cancer. HIF-1α was found to induce KLHL20 promoting the ubiquitination and degradation of PML (Yuan et al., 2011).

### Phosphorylation

Coordinated phosphorylation and isomerization appears to be a prerequisite for ubiquitin-mediated destruction of PML, a process involving a number of kinases. In response to hypoxia (as mentioned in see Ubiquitination), induction of KLHL20 by HIF-1α results in PML turnover. This requires the prior coordinated phosphorylation of PML by CDK1/2, followed by isomerization of the phosphorylated PML by the peptidyl-prolyl cis-trans isomerase, Pin1. This cascade is involved in cell transformation, migration, angiogenesis, and survival of mouse xenografts *in vivo*. Most importantly, the HIF-1α/KLHL20/Pin1 axis is upregulated in high grade aggressive and chemoresistant human prostate lesions and correlated with PML loss (Yuan et al., 2011).

Another example of this sequential PML preconditioning occurs with the extracellular signal regulated kinase 2 (ERK2), which is able to localize to the PML-NBs in breast cancer cells (MDA-MB-231), phosphorylate PML at two sites (S403 and S505), resulting in the recruitment of Pin1, and subsequent proteasomal degradation of PML by yet to be identified E3 ligase (Lim et al., 2011). Addition of hydrogen peroxide was capable of attenuating the association between PML and Pin1 to maintain PML levels. On the other hand, IGF-1 reduced PML levels in a Pin1 dependent manner, which enhanced cell migration (Reineke et al., 2010). Although ERK2 and Pin1 are frequently elevated in many cancers, the link to reduced PML levels *in vivo* is yet to be demonstrated.

Phosphorylation priming of PML has also been described without associated isomerization, however whether this second event is also important remains to be addressed. PML phosphorylation at multiple sites by casein kinase 2 (CK2), was shown by the elegant work of Scaglioni et al. (2006), to promote its proteasomal degradation, although the identity of the ubiquitin E3 ligase that is involved remains to be discovered. Further, analysis of NSCLC patient derived samples and cell lines, revealed that reduced PML levels directly correlated with increased CK2 activity, consistent with the relevance of this pathway to lung tumorigenesis (Scaglioni et al., 2006). Big MAP kinase 1 (BMK1) also phosphorylates PML at two sites: S403 and T409 (Yang et al., 2010). Mutational analysis demonstrated that BMK1 drives suppression of PML directly through its phosphorylation. Activation of BMK1 by its upstream MEK5 kinase results in the translocation of BMK1 from the cytosol to the PML-NBs (Yang et al., 2010). It was further demonstrated that activated BMK1 interferes with the formation of PML-Mdm2 complex, resulting in the suppression of p53 (Yang et al., 2012).

### Acetylation

The acetylation of PML represents an additional post-translation mechanism regulating PML. Treatment of HeLa cells with the HDAC (histone deacetylase) inhibitor, trichostatin A (TSA) resulted in increased acetylation of PML leading to efficient induction of apoptosis (Hayakawa et al., 2008). Importantly an acetylation-defective PML mutant renders cells refractory to HDAC inhibitor-induced cell death. The acetylation of PML could be enhanced by p300 acetylase. Interestingly the increase of PML acetylation was associated with the increase in the SUMOylation (Hayakawa et al., 2008). Hence it has been suggested that acetylation of PML may be a prerequisite for subsequent SUMOylation. It remains to be shown whether activation of PML by new generation HDAC inhibitors, currently under investigation, represents a key molecular event associated with clinical response.

### SUMOylation

The addition of small ubiquitin-like molecule (SUMO) to PML is essential for PML-NB formation and maturation, and may also mark PML for ubiquitination. SUMO may either be non-covalently bound to PML through the SUMO binding domain (Shen et al., 2006), or covalently attached by an E1, E2, and E3-ligase enzymatic cascade (Shen et al., 2006). PML SUMOylation also facilitates the recruitment of partner proteins to NBs and in turn their own SUMOylation (Shen et al., 2006; Bernardi and Pandolfi, 2007). Support of SUMOylation as key modification of PML is based on a number of studies. (Campagna et al., 2011) described a novel function for the histone deacetylase, SIRT1, in facilitating PML SUMOylation. The melanoma antigen gene A2, MageA2, interacts with PML isoform IV and significantly attenuates the SUMOylation and acetylation of PML, which in turn affects p53-mediated cellular senescence (Peche et al., 2012). The E3 SUMO ligase, protein inhibitor of activated STAT-1 (PIAS1), SUMOylates PML, and promotes the recruitment of CK2 to phosphorylate PML on S517 and consequently its degradation (Peche et al., 2012). PIAS1 regulates PML in NSCL cancer (Peche et al., 2012), and it has been implicated in As_2_O_3_-mediated degradation of PML-RARα in APL (Rabellino et al., 2012). Overall, the studies described above suggest a cascade of post-translational modifications involving phosphorylation, isomerization, and SUMOylation, regulating PML turnover (Chen et al., 2012b).

### Cytokine-dependent regulation of PML

There is growing evidence highlighting the importance of paracrine signaling in the regulation of PML whereby its transcription is controlled by interferons (IFNs), specific cytokines involved in anti-viral responses, immune-surveillance as well as anti-proliferative processes, which are known to be potent inducers of PML (Chelbi-Alix et al., 1995; Lavau et al., 1995; Stadler et al., 1995; Der et al., 1998). For example, IFNβ has been recently shown to induce cellular senescence, a key anti-cancer mechanism, via a PML-induced mechanism (Chiantore et al., 2012). In addition, genotoxic drugs such as etoposide trigger cellular senescence in normal and cancer cells via persistent activation of Janus kinase/signal transducer and activator of transcription (JAK/STAT) signaling and expression of IFN-stimulated genes including PML (Hubackova et al., 2010; Novakova et al., 2010). This is particularly interesting in the context of senescence-associated secretory phenotype (SASP), which constitutes an integral part of the paracrine/autocrine regulation of tumor suppression. Furthermore, PML was recently shown to be regulated by interleukin-6 (IL-6) through a molecular signaling pathway mediated by NFκB and JAK-STAT (Hubackova et al., 2012). Since IL-6 is a prototypical cytokine involved in cellular senescence, it is tempting to speculate that PML and IL-6 exist in a positive regulatory loop driving oncogenic and DNA-damage induced senescence. This would in turn lead to secretion of IL-6 and IFNs that would further sustain activation of PML.

## Therapies to Restore PML Tumor Suppression

A strategic approach to treating malignancies in which PML tumor suppressor activity has been reduced or lost through aberrant degradation, is to target those degradation pathways. However, in some contexts corrupted PML may provide a survival mechanism for disease and therapeutic benefit may result from its inhibition and will be discussed in Section “Therapies to Target Oncogenic PML Activities.”

### Therapies directed to inhibiting PML ubiquitination

**Figure 2:**
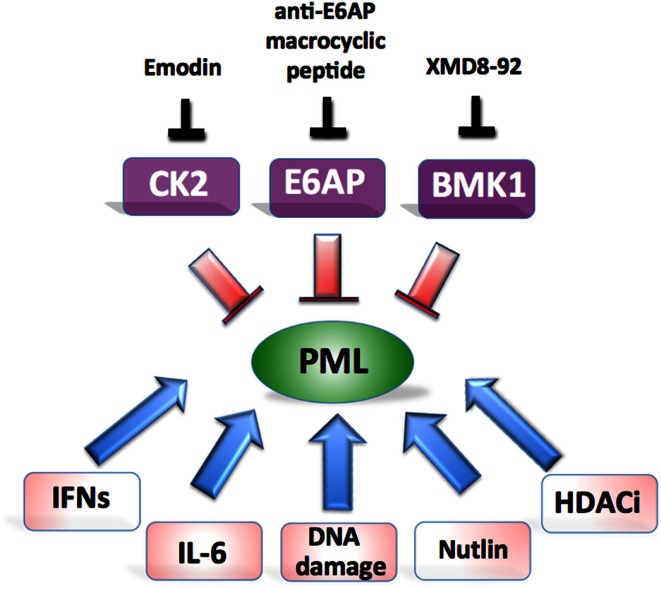
**Potential approaches aiming at restoration of PML-induced tumor suppression**. Major therapeutic targets and their inhibitors are shown on top and potential combinatorial treatments at the bottom.

In our recent study we demonstrated that E6AP elevation is frequently found in human B-cell lymphomas and is associated with PML downregulation (Wolyniec et al., 2012b). By using Myc-induced mouse model of lymphomagenesis and human B-cell lymphoma cell lines, we demonstrated that E6AP haploinsufficiency in mice or siRNA mediated inhibition of E6AP in human cells, is sufficient to restrain tumor development by inducing PML-dependent cellular senescence and preventing expansion of pre-leukemic B-cells. These observations provide the basis for considering E6AP as a promising anti-cancer target (Figure 2). Interestingly, a natural product-like macrocyclic *N*-methyl peptide inhibitor against E6AP has been synthesized by random non-standard peptides integrated discovery (RaPID) (Yamagishi et al., 2011). This peptide inhibitor, or other inhibitors of E6AP, can be used to test the possibility of relieving PML from proteasomal destruction in cancers where E6AP is elevated such as prostate adenocarcinoma and B-lymphoma (Srinivasan and Nawaz, 2011; Wolyniec et al., 2012b). Another therapeutic strategy could be to target Culin-3/KLHL20 mediated PML ubiquitination using a recently described Cullin-family E3 ligase inhibitor, MLN4924 (Soucy et al., 2009; Chen et al., 2012b). Although this inhibitor has been shown to exhibit potent anti-cancer activity by targeting NEDD8, it remains to be demonstrated whether PML is also involved in this mechanism.

### Therapy directed to inhibiting PML phosphorylation

Scaglioni et al. (2006) demonstrated that the reactivation of PML *in vivo* could be achieved in established lung cancer xenotransplants, with emodin, a pharmacological inhibitor of CK2 kinase (Figure 2). Although emodin exerts its effect through multiple pathways, this approach resulted in substantial suppression of growth that could be attributed to specific elevation of PML. Emodin treatment has previously been shown to increase the sensitivity of HeLa cells to As_2_O_3_ cytotoxicity through the generation of ROS, however the role of PML was not explored in this model (Yi et al., 2004).

Another very convincing strategy aimed at restoration of PML tumor suppression has been described in the elegant study by Yang et al. (2010) (Figure 2). By using a newly developed BMK1 inhibitor (XMD8-92), the authors were able to demonstrate efficient anti-tumor effect *in vitro* and in multiple xenotransplants *in vivo*, which was achieved by specific restoration of PML and downstream activation of p21. In their subsequent study they provided detailed explanation for the mechanism of BMK1 inhibitor by linking it to p53 activation (Yang et al., 2012).

### Combinatorial therapies to promote PML activation

Importantly, PML stability can be greatly enhanced by genotoxic drugs and HDAC inhibitors as well as cytokines such as IL-6 and IFNs. Therefore it is very likely that the right combinations of various PML activating strategies may translate to effective therapeutic outcomes (Figure 2). For example, treatment of cancer cells with etoposide triggers PML-induced senescence by engaging JAK/STAT pathway (Hubackova et al., 2010). This provides a rationale for a combination treatment of cancer cells with agents to stabilize PML such as emodin, XMD8-92, or anti-E6AP *N*-methyl peptide combined with DNA damaging agents and pro-senescence cytokines (Acosta and Gil, 2012). Such an approach should enhance cancer cell death but still requires formal testing. Our studies and others strongly support a link between PML and p53 whereby these proteins exist in a positive regulatory loop (Louria-Hayon et al., 2003; Bernardi et al., 2004; Alsheich-Bartok et al., 2008). Therefore the combined approach of restoring PML together with p53 (e.g., by using nutlin) should be tested. The tailoring of PML therapies to target multiple defined genetic malfunctions in individual cancers offers an exciting novel approach to inhibit cancer cell growth.

## Therapies to Target Oncogenic PML Activities

### Targeting PML-RARα to treat acute promyelocytic leukemia

Generation of the PML-RARα oncogenic fusion protein disrupts the normal functions of PML and RARα and is the driving pathogenic event in APL (de Thé et al., 1991; Rego et al., 2000; de Thé and Chen, 2010). PML-RARα impairs the assembly of PML-NBs and represses the expression of key regulatory genes involved in myeloid differentiation (Daniel et al., 1993; Zhu et al., 2002).

**Figure 3:**
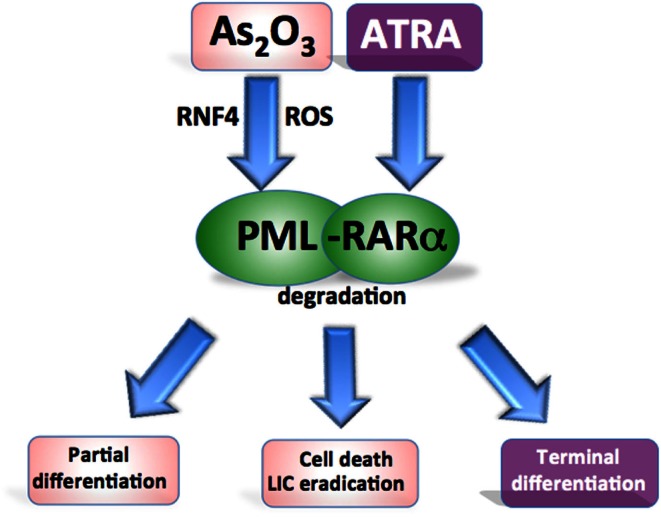
**Targeting oncogenic PML in acute promyelocytic leukemia**. Both agents target the PML-RARα fusion protein for degradation. ATRA promotes RARα-target gene transcription to overcome the differentiation block while As_2_O_3_ induces oxidant stress and directly binds PML to cause partial differentiation and apoptosis of APL cells and more effectively eradicate leukemia-initiating cells.

As_2_O_3_ and ATRA have been extensively used in the clinic as anti-APL therapy and have the overlapping effect of targeting and inducing the degradation of the PML-RARα fusion protein, thereby overcoming the differentiation block and restoring the senescence program in APL cells (Chen et al., 1997; Shao et al., 1998; Ferbeyre, 2002) (Figure 3). As_2_O_3_ is highly effective in the treatment APL and superior to ATRA in terms of its ability to achieve molecular remissions. As_2_O_3_ has been used as a single agent, and in combination with ATRA and chemotherapy achieving long term disease-free survival in up to 90% of APL patients (Hu et al., 2009; Ravandi et al., 2009; Mathews et al., 2010; Powell et al., 2010; Ghavamzadeh et al., 2011; Iland et al., 2012).

All-trans retinoic acid and As_2_O_3_ have the effect of restoring the normal distribution pattern of the PML associated NBs in APL (Koken et al., 1994; Weis et al., 1994; Muller et al., 1998b). In contrast to the NB reorganization induced by ATRA, As_2_O_3_ also causes swelling of the structures followed by a loss of PML staining. This may be the result of As_2_O_3_ inducing the attachment of multiple SUMO-1 molecules to PML (Muller et al., 1998a,b). As_2_O_3_ targets PML to induce degradation of both the PML-RARα fusion protein and PML (Lallemand-Breitenbach et al., 2012). The action of As_2_O_3_ is attributed to a combination of direct binding to PML and a more general oxidant effect (Jeanne et al., 2010; Zhang et al., 2010) (Figure 3). The inherent high ROS levels of APL cells contribute to their sensitivity to As_2_O_3_ (Yi et al., 2002; Li et al., 2008). Direct arsenic binding to PML results in topological changes in the RING domain enhancing the binding of the SUMO-conjugating enzyme (Jeanne et al., 2010; Zhang et al., 2010). As_2_O_3_ treatment also induces phosphorylation of the PML protein through a mitogen-activated protein (MAP) kinase pathway, which promotes efficient SUMOylation of PML (Hayakawa and Privalsky, 2004). Poly-SUMOylated PML is recognized by the SUMO-dependent ubiquitin ligase RNF4, poly-ubiquitinated, and degraded (Lallemand-Breitenbach et al., 2008; Tatham et al., 2008). Furthermore, As_2_O_3_ also induces apoptosis via oxidant stress (Miller et al., 2002).

### Targeting GOF PML activities in non-hematopoietic malignancies

Beyond APL, we have reported another example of what appears to be PML gain of function (GOF). In the background of mutant p53, PML tumor suppression cannot only be lost, but its activities can be conscripted to provide growth advantage. In fact, when PML was knocked down in these cancer cells they lapsed into growth arrest (Haupt et al., 2009). It is in this context that arsenic trioxide treatment of mutant p53 cancer cells is interesting, because although the drug has been demonstrated to target mutant p53 for proteasomal degradation (Yan et al., 2011), the activity against PML in this context has yet to be demonstrated, and neither has the consequence for cell viability.

Interestingly, PML was recently found to be elevated in a subpopulation of triple negative breast cancer patients and this correlated with reduced survival and poor prognosis (Carracedo et al., 2012). The functional studies revealed that in this context PML was able to simultaneously inhibit acetylation of peroxisome proliferator-activated receptor (PPARγ) co-activator 1A and activate PPAR signaling and fatty acid oxidation, which resulted in increased ATP. It will be critical to define the PML profile in these breast cancers where gain of pro-survival functions is apparent and fascinating to explore whether PML targeted therapy such as As_2_O_3_ has an application in these specific cancers. In addition, targeting the pathways activated by this elevated PML (i.e., fatty acid oxidation) may hold promise for therapy. However, targeting this pathway will need careful consideration as it is also involved in PML-dependent hematopoietic stem cell (HSC) maintenance (Ito et al., 2012).

### Targeting elevated levels of PML to eradicate leukemic stem cells

Promyelocytic leukemia plays a key role in the maintenance of HSCs and leukemia-initiating cells (LICs) (Ito et al., 2008). Murine Pml^−/−^ HSCs are not quiescent in the bone marrow of recipient mice and lacked long term repopulating capacity following transplantation. LICs share features of HSCs such as self-renewal, pluripotency, and quiescence (Reya et al., 2001; Hope et al., 2004; Holtz et al., 2007).

Quiescent chronic myeloid leukemia (CML) stem cells are resistant to conventional therapy including Bcr-Abl tyrosine kinase inhibitors and can be a source of relapse (Holtz et al., 2007). Unlike many other hematological malignancies, PML is highly expressed in CML particularly in more primitive CD34 positive cells (Ito et al., 2008). Indeed PML expression is an adverse prognostic factor in CML and being investigated as a potential therapeutic target in this disease. In a CML mouse model, Pml^−/−^ LICs undergo intensive cycling resulting in impairment of LIC maintenance and, in contrast to Pml wt cells, failed to initiate a CML-like disease after serial bone marrow transplantation procedures (Ito et al., 2008). By down-regulating PML, As_2_O_3_ also induces cell cycling of quiescent LICs and enhances cytarabine-mediated apoptosis to eradicate these cells in a serial transplantation model (Ito et al., 2008).

## Future Directions

Over the last decade, immense progress has been made regarding our understanding of molecular pathways regulating PML stability. Major post-translational modifications such as phosphorylation, acetylation, SUMOylation, and ubiquitination and their interactions have been extensively studied in normal and cancer cells. Although we have identified certain kinases and E3 ubiquitin ligases of PML, less is known about potential phosphatases and deubiquitinases, which are likely to play important regulatory functions. Another key limitation in our understanding of PML is the shortage of information about isoform specific effects and the potential problem with protecting and activating cancer promoting or cancer suppressing proteins. Hence, the new generation of mouse models specific for PML isoforms will be absolutely necessary to address these important issues. Treating of APL patients with As_2_O_3_ is usually very effective as it specifically targets the pathogenic PML-RARα fusion protein for degradation and reactivates functional PML. However, there is a proportion of APL patient that remains resistant and therefore novel therapies are required. Clearly, the restoration of PML to inhibit cancer growth emerges as a promising targeted strategy (Wolyniec et al., 2012a) that could be applied to many cancer types, given that PML plays a central tumor suppressive role in a wider range of human cancers than previously appreciated. Importantly it could be combined with currently used therapies such as chemotherapy, IFN, or IL-6 treatment, which are known to induce PML. Pertinently currently there are emerging several potentially druggable targets such as CK2, BMK1, KLHL20, and E6AP that has been demonstrated to negatively regulate PML stability in various pre-clinical cancer models. Further studies are required to evaluate the anti-cancer efficiency of the specific inhibitors of these molecules such as XMD8-92 in multiple pre-clinical models and eventually in clinical trials. However, there is a caveat concerning targeting of BMK1, related to the recent finding reporting that inhibition of BMK1 can stimulate EMT and cell migration (Chen et al., 2012a). As illustrated in this review one needs to carefully consider tissue type, genetic background, and stage of the disease in order to trigger PML for the benefit of patients with minimal possible side-effects. It becomes apparent that PML is regulated by different molecules in different cancer types, e.g., by E6AP in B-cell lymphoma (Wolyniec et al., 2012b), CK2, and PIAS1 in NSCL cancer (Scaglioni et al., 2006; Rabellino et al., 2012) whereas KLHL20/Pin1 in prostate adenocarcinoma (Yuan et al., 2011).

## Conflict of Interest Statement

The authors declare that the research was conducted in the absence of any commercial or financial relationships that could be construed as a potential conflict of interest.
